# Exploring patient-reported barriers to advance care planning in family practice

**DOI:** 10.1186/s12875-020-01167-0

**Published:** 2020-05-25

**Authors:** Carrie Bernard, Amy Tan, Marissa Slaven, Dawn Elston, Daren K. Heyland, Michelle Howard

**Affiliations:** 1 Department of Family Medicine, McMaster University, DBHSC 100 Main Street West, 5th floor, Hamilton, Ontario L8P 1H6 Canada; 2 Department of Family and Community Medicine, University of Toronto, Toronto, Ontario Canada; 3 Department of Family Medicine, University of Calgary, Calgary, Alberta Canada; 4 Division of Palliative Care, McMaster University, Hamilton, Ontario Canada; 5 Clinical Evaluation Research Unit, Kingston General Hospital, Kingston, Ontario Canada; 6 Department of Public Health, Queen’s University, Kingston, Ontario Canada; 7 Department of Critical Care Medicine, Kingston General Hospital, Kingston, Ontario Canada

**Keywords:** Primary care, Communication, Decision making, End-of-life, Older adults

## Abstract

**Background:**

Although patient-centred care has become increasingly important across all medical specialties, when it comes to end of life care, research has shown that treatments ordered are not often concordant with people’s expressed preferences. Patient and family engagement in Advance Care Planning (ACP) in the primary care setting could improve the concordance between patients’ wishes and the healthcare received when patients cannot speak for themselves. The aim of this study was to better understand the barriers faced by older patients regarding talking to their family members and family physicians about ACP.

**Methods:**

In this multi-site cross-sectional study, three free text questions regarding reasons patients found it difficult to discuss ACP with their families or their family physicians were part of a self-administered questionnaire about patients’ knowledge of and engagement in ACP. The questionnaire, which included closed ended questions followed by three probing open ended questions, was distributed in 20 family practices across 3 provinces in Canada. The free text responses were analyzed using thematic analysis and form the basis of this paper.

**Results:**

One hundred two participants provided an analyzable response to the survey when asked why they haven’t talked to someone about ACP. Two hundred fifty-four answered the question about talking to their physician and 340 answered the question about talking to family members. Eight distinct themes emerged from the free text response analysis: 1. They were too young for ACP; 2. The topic is too emotional; 3. The Medical Doctor (MD) should be responsible for bringing up ACP 4. A fear of negatively impacting the patient-physician relationship; 5. Not enough time in appointments; 6. Concern about family dynamics; 7. It’s not a priority; and 8. A lack of knowledge about ACP.

**Conclusions:**

Patients in our sample described many barriers to ACP discussions, including concerns about the effect these discussions may have on relationships with both family members and family physicians, and issues relating to patients’ knowledge and interpretation of the importance, responsibility for, or relevance of ACP itself. Family physicians may be uniquely placed to leverage the longitudinal, person- centred relationship they have with patients to mitigate some of these barriers.

## Background

Increasingly, medical specialties are focussing on patient-centredness as a key aspect of the care provided across the healthcare spectrum [1–3]. In a patient and family centred approach to care, emphasis is placed on collaboration, as healthcare is focused on an individual’s priorities, preferences, and values [1]. Family medicine has long prided itself in being a patient- centred profession, [4] always highlighting the need for putting the patient’s perspective and experiences front and centre in all healthcare conversations and decisions [3, 5, 6].

Although this approach seems noble, it appears to be mostly aspirational when it comes to communication and decision-making about future treatments and care at the end-of-life. Research has shown that the majority of seriously ill people approaching end-of-life have expressed a preference for non-invasive symptomatic treatment aimed at improving symptoms and quality of life [7] yet treatments ordered or received, are often not concordant with people’s preferences and are more aggressive than desired [8–10]. Some people may have considered what preferences they have regarding future and end-of-life care, but may not be able to communicate these preferences at the time decisions are needed [11, 12]. If they have not engaged in the process of advanced care planning (ACP) with family members and health care providers, these preferences cannot be known.

ACP is a communication process wherein people plan for a time when they cannot make decisions for themselves. It includes reflection, deliberation, and determination of a person’s values and wishes or preferences for future treatments and at the end-of-life. ACP should include communication between an individual, their family/loved ones, future substitute decision-maker(s) (SDM), and health care provider(s) about these values and wishes [13]. Prior engagement in ACP has been shown to improve patient and family experiences with healthcare near the end of life, resulting in greater concordance between patient wishes and the healthcare they receive, as well as lower stress and depression among family members [14, 15]. The public tends to think of ACP as only the process of creating a written advance directive for future health care [16]. These advance directives tend not to be used by clinicians, [17–19] and patients creating them tend to lack important knowledge relating to the treatment options described [20]. There is recognition that multiple behaviors are involved in successful ACP, notably conveying values and wishes to family members or friends even beyond the substitute decision-maker, and discussing wishes and future plans with health care professionals [21–23].

Patient and family engagement in ACP in the primary care setting could better prepare patients and families to make future healthcare decisions. In our previous research surveying patients in primary care about their ACP engagement, half of patients were aware of ACP and had talked to someone about it, however only 18% had talked to a health care professional [24]. Yet the process of ACP requires patients to integrate information about health conditions with their own values and preferences to ultimately be prepared for future decisions. The majority of Canadians have longitudinal relationships with their family physicians who provide care across the life cycle [25]. In addition, family physicians are well acquainted with their patients’ health conditions and needs and they care for their patients in the context of family units and support systems.

If we are to implement interventions to increase the uptake of ACP discussions as part of routine primary care, we need to understand the barriers patients face in having these discussions with their family members and family physicians [26]. As part of multi-site prospective audit of ACP in primary care settings, we conducted a content analysis of open-ended responses to a survey of patients regarding their engagement in ACP to gain a deeper understanding to the question: “What are the barriers faced by older adult patients regarding talking to their family members and family physicians about ACP?”

## Methods

### Setting and design

This multi-site cross-sectional study, conducted from October 2014 to March 2015 involved a self-administered questionnaire about patients’ knowledge of and engagement in ACP. The survey was conducted in a convenience sample of 20 family practices (i.e. with defined patient populations, not walk-in clinics), recruited by several of the investigators: thirteen from Ontario, five from Alberta, and two from British Columbia. Research assistants were present in the practices to provide participants with the questionnaires and to answer questions if they arose.

### Participants

Full details of the survey methods have been published previously [24]. Briefly, staff or clinicians were asked to invite consecutive eligible patients to participate on days the research assistant was present. Eligible patients were 50 years of age and older, could read and speak/understand English, and did not have a cognitive impairment. We chose the age of 50 based on literature suggesting the need for early engagement in ACP [27] and alignment with routine primary care delivery in this age group (e.g. screening) [28].

### Questionnaire

Details of questionnaire development and administration have previously been described [24]. The questionnaire asked whether patients had heard of ACP previously (a definition was provided), whether they had engaged in four key ACP behaviors including: 1) thought about the kinds of medical treatments they would want or not want if they were to get very sick and be in hospital, 2) talked to anyone about their wishes (and to whom), 3) written down their wishes, and 4) formally nominated a substitute decision maker, and finished questions about barriers to discussions.

Questions also included sociodemographic characteristics and a clinical frailty scale [29]. Patients self-completed the questionnaire. We report on the free-text responses to three questions:

1. If the respondent indicated they had not spoken with anyone about ACP:

“Why haven’t you talked with someone?”

2. “What is the one thing that makes it very hard for you to talk to your family doctor about medical treatments at the end of life?”
3. “What is the one thing that makes it very hard for you to talk to your family members about medical treatments at the end of life?”

### Analysis

Thematic analysis was used to analyze free text responses [30]. Five members of the research team (CB AT MS NS GA) independently reviewed the free text responses line by line and then categorized the emerging themes into groups in an iterative manner. The group then inductively developed a preliminary coding framework over multiple readings and discussions. These initial findings were discussed with the senior author (MH) to reach consensus on key themes and sub themes. These themes were reviewed by the research assistants (DE, NA) who had experience addressing any questions posed by the research participants while completing the survey. In a final meeting of the first two authors, the analysis was further refined through consensus and this analysis was presented to the remaining authors for review. The codes, themes, and subthemes were created and modified throughout the entire research process with full participation of 4 of the authors, ensuring that the rigor and trustworthiness of the data were maintained through a transparent and iterative process.

The study was approved by the research ethics boards of each participating academic institution. Research assistants received informed written consent at the time of administration.

## Results

### Participants

Most of the 20 participating family practices were group clinics and most employed allied health professionals as part of their team. Of the 878 patients who agreed to meet with the research assistant, 810 (92.2%) completed the survey. The mean age of patients was 66 years old (range 50–95 years) and 56% were female (440/809) (Table 1).

**Table 1:** Characteristics of the 810 patients from primary care practices

Characteristic	N (%)
Age, years, mean ± SD (minimum to maximum)	66 ± 10 (50 to 95)
Sex	
Female	450 (55.6)
Marital status	
Married or living as married	558 (69.0)
Widowed	100 (12.4)
Never married, divorced	151 (18.7)
Lives alone	179 (22.1)
Urban residence (self-defined)	718 (89.1)
Highest level of education	
Some or completed post-secondary	500 (61.9)
Spirituality or religion very or extremely important	365 (45.2)
Identifies with formal religious group or practice	
Protestant	295 (36.6)
Catholic	194 (24.1)
Other	136 (16.9)
None	180 (22.4)

Most patients identified as White/Caucasian (88%; 713/810) and the majority of patients (88%; 711/810) self-reported being ‘very fit’, ‘well’, or ‘managing well’ on the frailty scale. Four hundred thirty-nine respondents provided answers to the open ended questions. A total of 696 comments were analyzed.

### Thematic analysis

Approximately half (382/810; 47.2%) of respondents reported that they had not spoken with anyone about ACP. One-hundred two participants provided a response when asked why they haven’t talked to someone about ACP. Two hundred and fifty four provided a legible response to the question about talking to their physician and 340 provided a legible response to the question about talking to family members.

Eight distinct themes emerged from the free text response analysis (See Table 2).

**Table 2:** Themes and Illustrative Quotes

	Theme	Illustrative Quotes
**Across all three questions**	I’m too young/too healthy for ACP	“*Did not see this as necessary, it’s a bit soon. I am only 80!”* “I feel that I am too young for making those types of decisions” “not a priority as a healthy person” “I am not at that point in my life. If I was diagnosed with a terminal disease, then I would talk with him”
	Topic is too emotional	*“it’s a little frightening to talk about dying”* “sad emotional topic” “causing emotional pain to family” “Scares me; it is very negative and would depress me”
	ACP is the MD’s responsibility:	*“If a doctor wanted to talk to me about the end of life, it would not be hard for me to do”.* “He would have to suggest the discussion; MD should bring it up” “I would not want my family making medical decisions at the end of my life”
**Relating only to discussions with Family Physicians**	Fear of negative impact on relationship with MD	*I may be influenced to sway my thoughts”.* “Not knowing if his beliefs and values are the same as mine” “I am not sure that my doctor and I totally agree on treatments” “I don’t want to be unduly influenced by him”
	Not enough time in appointments	*“do not allow for an in depth conversation about something that is not happening now or may not ever happen. Appointments are too short”.* *“Doctor is always too busy. I feel I will impose on his time to talk about this when he has other patients waiting”.* “Family doctors are busy people and I would not consider it appropriate to ‘visit’ and discuss a broad topic like end of life”
**Relating only to discussions with family members**	Concern about family dynamics	“I don’t want arguments arising” “I don’t trust them very much to leave this kind of decision to them”
**Relating only to general questions about ACP discussions with anyone**	It’s not a priority	*“[I’m]too preoccupied”* “Though about it but just haven’t acted on it basically”
	I don’t know enough about ACP	*“Did not know about advance care planning; I think my family will know what to do”.*

#### Themes which emerged across all questions

##### A belief that the participant is too young/too healthy for ACP

Participants described feeling too young or too healthy to be thinking about ACP. Indeed participants seemed to believe that ACP should occur only after they were diagnosed with a life-limiting illness and that it was inappropriate to consider aspects of ACP when they perceived themselves to be ‘young’. Confusion around the age at which starting ACP might be appropriate was evident, with one participant writing, “*Did not see this as necessary, it’s a bit soon. I am only 80!”* (16–08-035, age 80, female).

##### The topic is too emotional for discussions

The fact that discussing ACP has the potential to bring up strong emotions was described as a deterrent to having the discussion. The topic was described as sad and depressing, and there was fear associated with discussing the end-of-life. It emerged that these conversations were perceived as being too emotional not only for the respondent (*“it’s a little frightening to talk about dying”)* (30–010-56 age 57, female) but also for the family, as participants reported being worried about *“causing emotional pain to family members”*. (16–04-036 age 52, female).

##### Believing that ACP is the MD’s responsibility (not the patient’s, not the family’s, the MD should bring it up)

Participants noted that someone else, other than the participant, should be primarily responsible for initiating ACP discussions. In response to the question about why participants did not discuss ACP with family members there was a clear theme that ACP should be the doctor’s responsibility, not the family’s. Participants indicated that although they felt comfortable discussing ACP with their MD, they saw it as the MD’s role to initiate and lead the discussion: *“If a doctor wanted to talk to me about the end of life, it would not be hard for me to do”.* (30–01-053, age 73, female).

#### Themes which emerged relating to discussions with family physicians

##### Fear of a potential negative impact on the relationship with the physician

It emerged that participants were concerned that having an ACP discussion might adversely affect the relationship with the doctor. One concern was that the doctor’s values may not align with the patient’s values, making the conversation difficult: “*Not knowing if his beliefs and values are the same as mine”.*(31–05-041, age 57, female) Other’s worried that the doctor may try to influence their decisions in a way that did not align with their wishes: *“I may be influenced to sway my thoughts* (11-01-011, age 59, female) Many respondents indicated they were concerned that it might be inappropriate to bring up such personal issues in a doctor’s appointment *“it’s a personal family matter”*. (20–06-019, age 58, female).

##### Not enough time in the appointments

Insufficient time in appointments with family physicians emerged as a barrier to ACP discussions. Participants reported that there were significant time constraints on appointments which *“do not allow for an in depth conversation about something that is not happening now or may not ever happen. Appointments are too short”.* (16–06-037, age 55, female) Further, they seemed to worry about whether this type of appointment may take time away from others: *“Doctor is always too busy. I feel I will impose on his time to talk about this when he has other patients waiting”.* (19–01-006, age 59, female).

#### Themes which emerged relating only to discussions with family members

##### Concern about family dynamics

Participants expressed concern about how an ACP discussion may affect family dynamics. Participants described a worry that such a discussion would cause conflict within the family and they wanted to avoid such conflict: *“I don’t want any arguments arising”.* (30–03-094, age 65, female) Analysis brought forth concerns about how discussions might affect one’s spouse, or one’s children. Some participants did not want to discuss ACP because of dynamics and lack of trust among family members, which may affect the ACP discussion: “*I don’t trust them very much to leave this kind of decision to them”.* (19–01-010, age 58, female).

#### Themes which emerged relating to the general question about ACP discussions with anyone

##### It’s not a priority

A key theme that ACP was not a priority for participants arose. This is exemplified by quotes such as: *“[I’m] too preoccupied”* (16–08-014, age 52, female) and *“[I have] thought about it but just haven’t acted on it yet”.* (31–01-030, age 62, male).

##### I don’t know enough about ACP

When participants explained that they did not know about ACP, many of them went on to add that they felt that someone else would take care of it anyway *“Did not know about [advance] care planning; I think my family will know what to do”.* (11–01-023 age 54, female).

## Discussion

Through qualitative analysis of free text responses to open ended questions about what makes it difficult to have ACP discussions, we found several overall themes relating to patients’ ACP discussions with physicians and family members. The emergent themes included barriers as perceived by the patients to ACP discussions, concerns patients have about the effect these discussions may have on relationships and dynamics with both family members and family physicians, and issues relating to patients’ knowledge and interpretation of the importance, responsibility for, or relevance of ACP itself.

The centrality of the unique patient-family physician relationship has the potential to mitigate some of the challenges to advance care planning we uncovered in our study. Similar to previous literature, [31, 32] our study indicates that even when patients are indeed ready to have a conversation about ACP, they often expect their family doctor to let them know ***when*** to broach the topic. Patients who believe that they are “too young” or “too healthy” may not understand that ACP is necessary to prepare their SDM to speak for them at **any** point when they may not be able to speak for themselves; this may not be in the context of end of life care [21, 22, 33]. Thus, it seems that it is essential that family doctors take responsibility for initiating these discussions and for educating patients about the importance of ACP in many different circumstances.

Although in our study patients expressed an expectation that their physician would let them know when to discuss ACP, in a previous study some patients in primary care felt they, themselves, should initiate the discussion [34]. Even when this is the case, patients who believe that they are “too young” or “too healthy” for ACP may not initiate ACP conversations early enough to be of benefit. Given that family physicians regularly engage in proactive medicine and bring up issues which patients do not bring up themselves as a usual part of care, including ACP as a routine topic in preventive care and screening discussions would be an appropriate place to start [35, 36]. Our findings may empower family physicians who believe that patients or families should initiate ACP discussions [37] to instead make the first move and introduce ACP to their patients. Education on effective resources already developed, such as the “Just Ask” tool [38] and “Speak Up” [38, 39] as part of the Canadian Advance Care Planning website (http://www.advancecareplanning.ca/resource/just-ask/), and the Plan Well Guide**™** website (https://planwellguide.com/) are tools that can both remind and facilitate family physicians in their efforts to initiate these conversations with their patients. Validated communication tools, such as the Serious Illness Conversation guide, can help family physicians prepare for ACP discussions while training sessions using this guide can help to build confidence in these skills [40, 41].

Family physicians also care for their patients within the context of their families, sometimes caring for several family members in one family, and attend to patients’ physical and emotional needs. Helping a patient to navigate through the fears and worries identified in our study regarding discussing ACP, either by working with the patient in the context of their family or by engaging other members of the health care team is a meaningful contribution that most family physicians could fulfill. As noted above, by educating patients on the misconception that ACP is only related to end-of-life decision-making, family physicians can help quell the heightened emotions that patients may fear will arise in ACP discussion. Further, helping patients navigate through the purposeful reflection of their values and wishes with regards to care can be empowering for patients.

Previous work revealed that patients did not perceive ACP as relevant to them because they were healthy [21] and that the topic brings up difficult emotions among people with whom they have relationships such as family [21, 42, 43]. In addition, the belief that patients’ family members will know the patient’s wishes is not uncommon [16, 42] despite evidence that family carers are often unaware of patient preferences [44]. In addition, the presence of decisional conflict and regret is known to be high in family members of critically ill patients [45] and negative emotional burden [46] and burden of decision-making is lower when ACP has been undertaken [47]. Our study shows similar findings but provides new insights into the role of family physicians in engaging and educating patients about the importance of ACP as it may pertain to the involvement of others in decisions about their health care. Having these conversations in the context of routine preventive visits may help to change culture. Patients will hopefully see choosing an SDM and sharing goals and wishes as something usual and necessary to prepare for any time where a patient may lose the capacity to participate in decision-making and not related only to end of life care. Public health campaigns highlighting the 2017 consensus definition of ACP [22] could help the population as a whole to better understand the importance ACP for all adults. Further, materials aimed at engaging patients could be available in family medicine offices, encouraging patients to be ready for such discussions with family physicians as a usual aspect of care for all adults. For example, when older adults were randomized to self-directed use of an interactive website to engage in ACP that explains the importance of asking doctors questions, they were more likely to raise the issue of ACP with their primary care provider compared to people who were given an advance directive form only [48].

Both patients and family physicians are aware of how current clinical care and relationships are influenced by time pressures and conflicting priorities. Family doctors are aware that more time is needed to allow for ACP discussions [37, 49–51]. Indeed time has been described as the “ultimate barrier” to creating the best possible circumstances for a “good death” in one study of family physicians’ experiences of conflict with substitute decision-makers [52]. Given the ongoing longitudinal relationships that typically exist between family physicians and their patients (in the Canadian context), [53] seeing ACP as a process as opposed to a one-time conversation could help to manage the time-related pressures perceived in the context of individual appointments. Given the evidence that patients can be engaged in ACP, funding models need to be adjusted to enable family physicians to spend the time needed for ACP discussions [52] Further, other, non-physician health care providers can play an important role in ACP discussions, [54–57] but the specifics of how and when they are involved will depend upon the organizational structures and funding models in various family physicians’ clinics.. These efforts could consider models like the Respecting Choices® program which aim to engage individuals across the age and health spectrum in ACP and utilizes non-physician facilitators as a part of the ACP process [58]. Such efforts may better prepare patients to have discussions with their family physicians regarding ACP and their specific health situation, reducing the need for multiple time- intensive visits.

The finding that patients were concerned about how an ACP discussion might affect their relationship with their family physician suggests that efforts are needed to normalize such discussions as part of routine clinical care for the patient and for the practitioner. We found that patients worry about disagreeing with the family doctor, being ‘unduly influenced’ by an ACP discussion, and taking too much of the ‘doctor’s time’. This stands in stark contrast to the commitment to patient-centredness which rests at the core of the doctor-patient relationship in family medicine [4, 6]. Family physicians may need to be attuned to these concerns expressed by patients and reassure patients of the advisory role that the family physician plays in facilitating patients to make fully informed decisions that are in keeping with the ***patient’s*** own values and goals.

### Strengths and limitations

A strength of this study is that it was conducted across multiple family practices and geographies. The free text nature of our questions allowed for additional information to be gathered to elaborate on the previously reported categorical responses to questions about ACP behaviours [24]. The themes derived from the data could guide the creation of closed-ended response options in future questionnaires assessing ACP engagement and barriers.

Our study also has limitations. Not all participants provided responses to the open-ended questions and we cannot be certain that the results would represent the views of all participants. In addition, participants self-completed the survey and we were unable to ask for clarification of ideas expressed and most responses were short. The participating practices and patients were not randomly selected, which may reduce generalizability. In addition, the patients were required to speak and understand English, and only 12% identified themselves as other than white/Caucasian, which is not representative of the contemporary Canadian population [59].

Finally, the definition of ACP which was provided to patients “reflection, deliberation, and determination of a person’s values and wishes or preferences for future treatments at the **end-of-life**” may have directed their attention towards considering ACP as something one **only** considers near the end of life. The newer consensus definition from 2017: “Advance care planning is a process that supports adults at any age or stage of health in understanding and sharing their personal values, life goals, and preferences regarding future medical care” [22] may have yielded different responses.

## Conclusion

Our findings indicate that lack of awareness about ACP and not seeing ACP as a priority were key challenges among patients who reported a barrier to ACP discussions. Further, even when aware of the importance of ACP, patients may not feel that it is their role to initiate the discussion with their physician or family members. It may be possible to address this reluctance through the unique longitudinal relationship that family physicians have with their patients and through a focused effort to bring the ACP conversation into the forefront of routine preventive care. With better patient education, a preventive focus situated in the ongoing longitudinal family physician-patient relationship, and appropriate funding models, ACP discussions could happen at appropriate times (e.g. after hospitalizations, when health status undergoes a significant change, at routine visits for older adults) and could help patients receive the care that respects their goals and values in their future care or if they are not able to speak for themselves.

## Data Availability

The datasets used and/or analysed during the current study available from the corresponding author on reasonable request..

## Abbreviations

ACP: Advance Care Planning
MD: Medical Doctor
SDM: Substitute Decision Maker
